# Phenotype-Genotype Discordance and a Case of a Disorder of Sexual Differentiation

**DOI:** 10.1155/2024/9936936

**Published:** 2024-07-17

**Authors:** Madeline Snipes, Stephanie Stokes, Amy Vidalin, Lee D. Moore, Natalia Schlabritz-Lutsevich, James Maher

**Affiliations:** ^1^Augusta University, Department of Obstetrics and Gynecology, Augusta, GA, USA; ^2^Texas Tech University Health Science Center, Permian Basin, Odessa, TX, USA; ^3^Advanced Fertility Centers, Odessa, TX, USA

## Abstract

Discordance between the genetic sex and phenotype seen on ultrasound can identify disorders of sexual development (DSD) that previously escaped detection until puberty. We describe a 46, XY disorder of sexual differentiation caused by a rare mutation in the *SF1* gene (OMIM]184757, (*NR5A1*). The mutation (*NR5A1*)-c.205C > G (p. Arg69Gly) was discovered after a phenotype-genotype discrepancy was encountered during prenatal care. The baby with 46, XY DSD has female external genitalia but evidence of Y chromosome-related regression of Müllerian structures and the absence of palpable gonads. We discussed the literature on phenotype-genotype discrepancy and the importance of care coordination between the antenatal and postnatal teams to ensure a timely diagnosis of DSD.

## 1. Introduction

Cell-free DNA testing has changed prenatal screening for common aneuploidies. It is now possible to disclose the fetal sex as early as ten weeks gestation. Discordance between the genetic sex and the subsequent phenotype seen on ultrasound will identify rare disorders of sexual development that previously escaped detection until puberty [1–3]. There are several potential clinical explanations for discordance between noninvasive prenatal testing by cell-free DNA and ultrasound evaluation of fetal sex, which range from human error in sample labeling, laboratory evaluation, or ultrasound performance, to rare genetic abnormalities, including disorders of sexual differentiation [4, 5].

In 2015, Bianchi et al. evaluated the results of genotype-phenotype discordance, including fetal sex [6]. They recommended evaluation for disorders of sexual differentiation (DSD) if the workup did not reveal another etiology for the discordance [6]. Smet et al. reviewed phenotype-genotype discordance and offered an algorithm to approach the antenatal workup and diagnosis [4]. Richardson et al. estimated the discordance rate at between one and 1,500 to 2000 pregnancies [5] and emphasized the importance of good communication between the obstetrician, pediatric providers, parents, and postnatal care providers when disorders of sexual differentiation are suspected [5].

Dhamankar et al. reported 91 cases of discordance between the cell-free-DNA results and the clinical or ultrasound evaluation of fetal sex in 1.3 million samples analyzed [7]. Eighty-three patients had sufficient clinical information to allow outcome assignment, and 36% of the discordance was due to disorders of sexual differentiation—a further 34% were related to human or methodological error.

Recently, De Falco et al. described trio testing in a case with phenotype-genotype discordance between the cell-free DNA and ultrasound results. Exome sequencing on cell-free fetal DNA and parental DNA identified a previously unsuspected heterozygous mutation in the *HSD 17 B3* gene, subsequently confirmed on amniocentesis [8].

The diagnosis of a DSD can create confusion; the language used to relay the information affects acceptance of the diagnosis, including the rate of pregnancy continuation. The ACCORD alliance offers examples of information in a standardized approach that provides the family with evidence-based information. Other resources include dsdfamilies (https://www.dsdfamilies.org) andhttps://www.steroidogenicfactor1.info [9].

Here, we describe a 46, XY disorder of sexual differentiation caused by a rare mutation in the *SF1*gene after identifying a discrepancy between cell-free DNA and ultrasound.

## 2. Case Description

A 21-year-old G1P0 female presented for MFM consultation regarding discordance between NIPT and anatomy ultrasound. NIPT reported her fetus as a low-risk male at 12 weeks gestation; an anatomy ultrasound at 20 weeks demonstrated female external genitalia. The patient received counseling regarding this discrepancy and underwent amniocentesis, confirming a 46, XY genotype with a positive SRY gene.

The patient continued regular prenatal appointments and presented in spontaneous labor at 37.3 weeks. The infant was born via uncomplicated vaginal delivery, with a birth weight of 2790 g and APGAR scores of 7 and 8 at 1 and 5 minutes, respectively. The infant appeared phenotypically female with mild posterior labial fusion, an open introitus, no palpable gonads, and standard clitoral size at the confluence of the labia minora.

Pelvic and retroperitoneal ultrasound demonstrated no uterus or ovaries. The left kidney was found in the renal fossa. A right kidney was reported to be ectopically located in the pelvis.

Pediatric endocrinology is following the child. The neonatal FSH was 20.09 mIU/mL, LH was 1.65 mIU/mL, testosterone was 48.96 ng/dL, estradiol was <15 pg/mL, and a 53 gene panel from Invitae testing for disorders of sexual differentiation resulted in a negative screen for known pathogenic mutations. The analysis revealed two mutations reported as “variance of unknown significance.” The first mutation detected was within the *Aristaless-related homeobox* gene *(ARX)*−196 + 6G > A. The second was within the steroidogenic factor one gene, also known as the NR five A1 nuclear receptor *(NR5A1)* − *(OMIM 184757)*c.205C > G (p. Arg69Gly). The SRY gene was not affected. Inhibin and anti-Müllerian hormone levels were not assessed.

The family is currently giving the child a female gender assignment. The patient continues to follow up with pediatric endocrinology, urology, and the general pediatrician. Exam under anesthesia, laparoscopy, and cystourethroscopy at 20 months demonstrated mild clitoromegaly, blind-ending vaginal pouch, normal-appearing bilateral testes in the abdomen proximal to the inguinal ring, absent uterus and upper vagina, and normal bladder and ureteric orifices. A right testicular biopsy was performed without the need for gonadectomy due to normal appearance. The biopsy showed seminiferous tubules containing Sertoli cells only. No germ cells were seen on H&E or immunohistochemical staining.

## 3. Discussion

The discrepancy between the phenotypic sex on ultrasound and the genotypic sex on NIPT or karyotype has several potential methodological and biological explanations. These include human error in sample labeling, lab error, ultrasound error in assigning gender, maternal origins including transplanted organs from the opposite sex, maternal or placental chimeras, maternal neoplasm, placental mosaicism, co-twin demise, and DSD [2, 4, 10, 11]. After excluding the common explanations for discordance, invasive testing should be offered to confirm the genetic sex and evaluate for the presence of the SRY gene and pathogenic copy number variants on a microarray. A gene panel can look for common pathogenic mutations when a DSD is suspected [12].

Disorders of sexual differentiation are broadly categorized into three main categories in the Chicago Consensus: sex chromosome DSD; 46, XY DSD; and 46, XX DSD [13, 14]. Each will include a unique grouping of etiologies.

While some cases of 46, XY DSD were diagnosed due to a family history of a prior affected individual or because of the evaluation of ambiguous genitalia at birth, many cases that occurred before the widespread adoption of molecular testing were found after the affected individual presented in the assessment of delayed puberty, primary infertility, amenorrhea, or virilization at puberty. 46, XY DSD is a heterogeneous group of congenital conditions with a variable degree of virilization of the external genitalia and a spectrum of Wolffian and Müllerian duct structural development [15]. Some cases of 46, XY DSD are monogenic, while others are either oligogenic or the result of a complex interaction among multiple genes and target organs [16]. Testicular tissue is identified in many but not all patients, regardless of the degree of virilization. Complete absence of virilization results in female external genitalia and, until recently, would escape detection until puberty.

The management of an infant with a known or suspected DSD requires a multidisciplinary team to address the complex issues in the child's care. Physical exam, imaging, hormonal, and electrolyte analysis are employed to rule out life-threatening conditions such as renal or adrenal failure as well as Wilms tumor. Genetic evaluation can often arrive at a precise molecular diagnosis. The analysis includes DSD gene panels and, if needed, exome or transcriptome evaluation. This information can then lead to a comprehensive treatment plan, including topics such as gender assignment and sex of rearing, as well as clinical, psychological, and long-term endocrine follow-up [17].

In the past, patients with 46, XY DSD were classified into broad groupings based on clinical, hormonal, and imaging studies such as gonadal dysgenesis, disorders of androgen and anti-Müllerian hormone secretion or action, or DSD of unknown etiology [13, 18]. The specific diagnosis of 46, XY DSD is often more challenging. MPS technology has advanced the diagnostic yield by allowing the investigation of multiple target genes. The combined approach of clinical, biochemical, and genetic analysis is statistically superior to either approach alone in arriving at the correct diagnosis [19].

Steroidogenic factor 1 (*SF1*, *NR5A1*) is a nuclear receptor regulator that coordinates the action of multiple genes involved in embryonic adrenal and gonadal formation, steroid production, and reproductive development. In humans, SF1 mutations were first described in two patients with 46, XY disorders of sex development (DSD) who presented with adrenal failure and gonadal dysgenesis with persistent Müllerian derivatives. (OMIM]184757 [20]. Mutations in *NR5A1* are among the most frequently identified genetic causes of gonadal development disorders and are associated with a wide phenotypic spectrum. *NR5A1*-related phenotypes have a wide range of findings in both sexes. In males, it may range from disorders of sex development (DSD) with ambiguous or even female external genitalia to oligo/azoospermia in a phenotypically normal male. In 46, XX female individuals, the phenotype can range from 46, XX Ovo testicular and testicular DSD to primary ovarian insufficiency (POI) [9, 21]. Variants in the *NR5A1* gene are the most frequent genetic cause of DSD in 46, XY patients [19], accounting for 10–20% of cases of 46, XY DSD [22]. Phenotypes vary from males with spontaneous puberty, substantial testosterone production, and possible fertility to females with and without Müllerian structures and primary amenorrhea. Müllerian derivatives are present in about 24% of the cases [23]. Mothers and sisters of 46, XY DSD patients carrying heterozygous *NR5A1* mutations may also have the mutation and develop POI [24].

SF-1 is expressed in the bipotential embryonic gonad and regulates the differentiation towards testes through genes like SRY and SOX9 [25]. SF-1 is also involved in the regression of the paramesonephric duct via anti-Müllerian hormone (AMH) in Sertoli cells and the virilization by regulation of the biosynthesis of testosterone in Leydig cells [26, 27]. Postnatally, Leydig cell's function seems to be preserved in many patients with *NR5A1* mutations. In contrast, Sertoli and germ cells are more profoundly affected. The external genitalia often appear underdeveloped at birth, but may undergo spontaneous virilization during puberty due to these mutations.

Phenotypic variability observed in patients with similar *NR5A1* mutations may be related to the presence of other associated epigenetic modifiers or coinheritance of pathogenic variants in different testis/ovarian-determining genes [13, 16, 24, 28]. Even with the same family, the identical mutation has been associated with different phenotypes [24]. In a study by Song et al., the phenotype of 30 children with 46, XY DSDs with *NR5A1* mutations was analyzed [29]. All of the patients in the study had testes, and none had a uterus or ovaries; thirteen patients had inguinal testes. Domenice et al. found the most common clinical presentation to be “atypical or female external genitalia with clitoromegaly, palpable gonads, and absence of Müllerian derivatives [13].” Currently, there are over 200 known pathogenic variants in the *NR5A1* gene reported in association with individuals with DSD [13, 30–36].

The NSR5A1 mutation (NR5A1)-c.205C > G (p. Arg69Gly) seen in our patient was previously reported in a 5-year-old girl who was evaluated for clitoromegaly and found to have atrophic testicular tissue in the abdomen [37]. Although this variant was listed as having unknown significance, our finding of a similar phenotype with the same mutation increases the likelihood that the variant is pathogenic and etiologic in this child's condition. This mutation was also seen in a Chinese child with a bifid scrotum, a small penis, and no uterus [24]. Invitae lists this mutation as “Heterozygous, Uncertain Significance.” However, the mutation replaces Arginine, which is basic and polar, with Glycine, which is nonpolar at codon 69 of the protein within the Zinc Finger [9, 38]. Western blot assay showed that this mutation is associated with severely lowered protein expression compared to the wild-type protein SF1 expression [24]. Overall, SIFT predicts this mutation as damaging, PolyPhen-2 predicts that it is probably damaging, MutationTaster predicts that it is disease-causing, and ACMG would classify it as likely pathogenic [13].

The child also has a mutation in the Aristaless-related homeobox gene, ARX, which is an important transcription factor for the developing forebrain, pancreas, and testes [39]. Although our patient's mutation is intronic, it affects nucleotides within the consensus splice site, a common cause of aberrant splicing. Many investigators have noted the wide phenotype spectrum and poor genotype-phenotype correlation in similar NR5A1 mutations and speculate that this variability results from the contribution of other genetic modifiers [16, 28]. Exome sequencing analyses of DSD patients can identify pathogenic variants or variants of uncertain significance in several genes involved in sexual development in patients with an NR5A1 mutation.

Byers et al. discuss the ethical implications of DSD and emphasize the importance of regular follow-up with a multidisciplinary team comprised of a general pediatrician, urologist, psychiatrist, and endocrinologist beginning as soon after birth as possible [16, 40, 41]. The ACCORD alliance is a valuable resource for families and caregivers. Topics that need to be addressed with these children as they age include gender dysphoria, infertility, risk of malignancy, osteoporosis, the need for hormone replacement, virilization, and the potential need for genitoplasty.

In conclusion, we report a case of phenotype-genotype discordance between genetic sex and the eventual postnatal appearance of the external genitalia of the infant. This child was subsequently found to have two novel genetic mutations linked to sexual differentiation. We discuss the literature on phenotype-genotype discrepancy and the importance of care coordination between obstetrics and pediatrics to ensure a timely diagnosis of DSD. Our case demonstrates a baby with 46, XY DSD and female external genitalia but evidence of Y chromosome-related regression of Müllerian structures and the absence of palpable gonads. Sequence analysis and duplication/deletion testing by Invitae using the 53 gene disorder of sex development panel revealed mutations within the *Aristaless-related homeobox gene (ARX)* −196 + 6G > A and steroidogenic factor one NR five A1 nuclear receptor *(NR5A1)-*c.205C > G (p. Arg69Gly). They were likely etiologic in the development of the DSD. The widespread adoption of cell-free DNA testing will increase the detection of rare cases of DSD.

## Data Availability

The data are provided within the body of the paper.
